# Key Technologies of Pure Hydrogen and Hydrogen-Mixed Natural Gas Pipeline Transportation

**DOI:** 10.1021/acsomega.3c01131

**Published:** 2023-05-19

**Authors:** Chaoyang Zhang, Yanbo Shao, Wenpeng Shen, Hao Li, Zilong Nan, Meiqin Dong, Jiang Bian, Xuewen Cao

**Affiliations:** † China Petroleum Engineering & Construction Corporation North China Company, Renqiu 061000, People’s Republic of China; ‡ College of Pipeline and Civil Engineering, China University of Petroleum (East China), Qingdao 266580, People’s Republic of China; § PipeChina Engineering Technology Innovation Co., Ltd., Tianjin 300450, People’s Republic of China

## Abstract

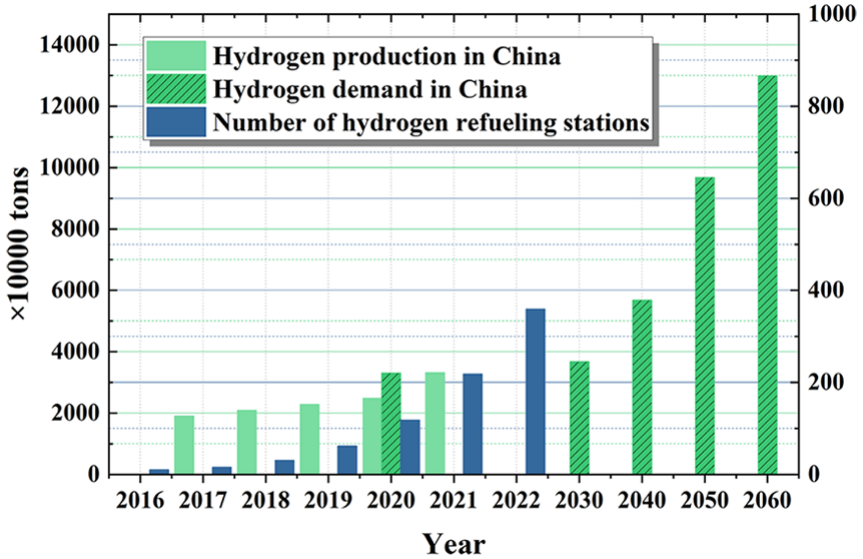

Thanks to the advantages of cleanliness, high efficiency, extensive sources, and renewable energy, hydrogen energy has gradually become the focus of energy development in the world’s major economies. At present, the natural gas transportation pipeline network is relatively complete, while hydrogen transportation technology faces many challenges, such as the lack of technical specifications, high safety risks, and high investment costs, which are the key factors that hinder the development of hydrogen pipeline transportation. This paper provides a comprehensive overview and summary of the current status and development prospects of pure hydrogen and hydrogen-mixed natural gas pipeline transportation. Analysts believe that basic studies and case studies for hydrogen infrastructure transformation and system optimization have received extensive attention, and related technical studies are mainly focused on pipeline transportation processes, pipe evaluation, and safe operation guarantees. There are still technical challenges in hydrogen-mixed natural gas pipelines in terms of the doping ratio and hydrogen separation and purification. To promote the industrial application of hydrogen energy, it is necessary to develop more efficient, low-cost, and low-energy-consumption hydrogen storage materials.

## 1 Introduction

Global warming and energy crises are among the most important issues that threaten the peaceful existence of human beings. Climate and energy issues have been attracting attention over the past century, but it is only in recent years that nations have developed general solutions.^1,2^ Many researchers have investigated these issues and generally agree that the use of clean energy and carbon-emission-free renewable energy is the key to solving energy and climate problems.^3^ After the global energy crisis in 1974, the idea of using hydrogen as a source of energy became popular with researchers.^4^ The outstanding nature and characteristics of hydrogen make it a very promising energy source. Hydrogen is abundant in reserves, with a wide range of sources,^5^ high energy per mass, and reduced carbon emissions.^6^

For many researchers studying hydrogen storage and transport, there is a need to acquire extensive knowledge about the various processes involved in hydrogen storage and transportation, as well as their advantages, disadvantages, and aspects to be optimized to make hydrogen more suitable for future developments. There are few related articles, and most papers only address certain specific aspects of the topic. In this paper, the current state of research and application of existing technologies in various aspects of hydrogen storage and transportation are analyzed. The focus is on the introduction and summary of hydrogen pipeline transportation and hydrogen-mixed natural gas pipeline transportation.

## 2 Hydrogen Storage and Transportation Technologies

**Figure 1:**
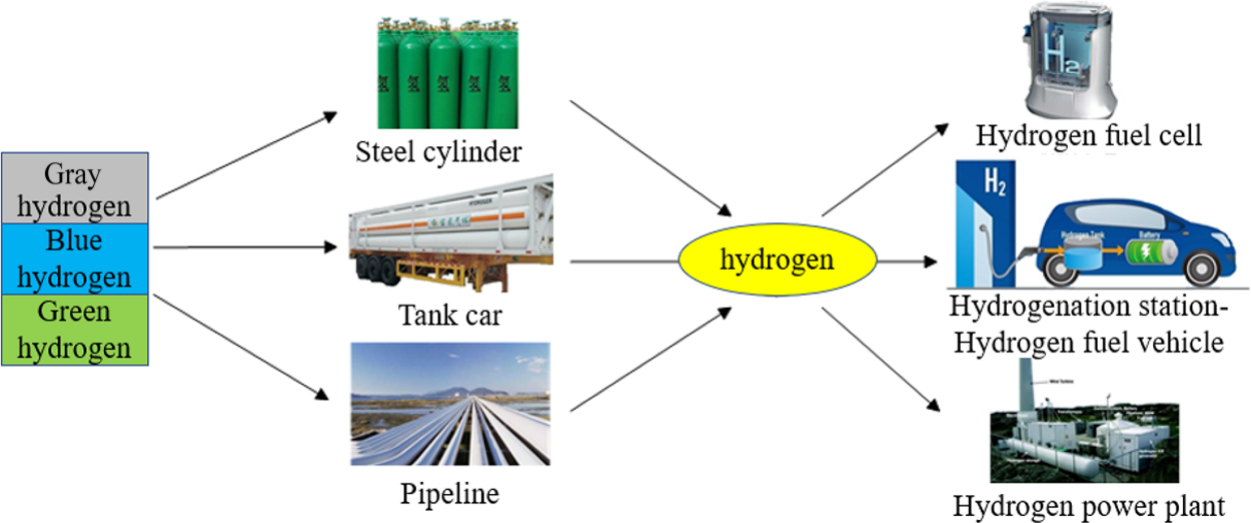
Overview of the hydrogen industry chain.

Hydrogen produced through the combustion of fossil fuels (such as oil, natural gas, coal, etc.) is called gray hydrogen, and the production of gray hydrogen is associated with emissions such as carbon dioxide. Hydrogen made from natural gas by steam methane reforming or autothermal steam reforming is called blue hydrogen.^7,8^ Although natural gas is also a fossil fuel and greenhouse gases are produced in the production of blue hydrogen, greenhouse gases are captured due to the use of advanced technologies such as carbon capture, utilization, and storage (CCUS), which reduces the impact on the global environment and enables low-emissions production. Hydrogen produced by using renewable energy sources (such as solar,^9^ wind,^10^ nuclear,^11^ etc.) is called green hydrogen, such as hydrogen produced by the electrolysis of water through renewable energy generation, and there are no carbon emissions in the production of green hydrogen.^12^ Green hydrogen is the ideal form of hydrogen energy utilization, but it will take time to achieve large-scale application due to the current technology and manufacturing cost limitations.^13^ An overview of the hydrogen industry chain is shown in Figure 1.

Hydrogen storage and transportation mainly include high-pressure hydrogen gas storage and transportation, liquid hydrogen storage and transportation, and solid-state hydrogen storage and transportation (Table 1). In addition, liquid organic hydrogen carrier (LOHC) transportation is a new type of liquid storage and transportation technology that has emerged in recent years.^14^ It and solid-state hydrogen storage technology are gradually gaining traction among researchers.^15^

**Table 1:** Overview of Hydrogen Storage and Transportation Technologies^1,24,49^

status	carrier	capacity range	applications, advantages, and disadvantages	unit cost^a^
high-pressure hydrogen gas	sealed container			
	bundle	5–10 kg/grid	more mature, small transport volumes, short transport distances	★★★
	integrated pipe	250–460 kg/vehicle	medium and long distance transportation, widely used	
	pipeline	310–8900 kg/h	long-distance transportation, large throughput and high efficiency, the natural gas pipeline network can be used to reduce initial investment.	★★★☆
liquid hydrogen	sealed container			
	tanker truck	3–80 m^3^/ vehicle	high energy consumption and high technical requirements for liquefaction	★☆
	railway	100–200 m^3^/tank	high transport volume and high energy consumption for liquefaction	
	barge	up to 1000 m^3^/tank	high transport volume, high energy consumption for liquefaction, economical, and safe	
	liquid organic hydrogen carrier (LOHC)		in the testing stage, the hydrogen storage density is large and the cost is high, high security, high development potential	★★★★ R&D costs may be high for the moment
	pipeline		the cost is high and the technology is difficult; at present, it is only used for short distance transportation or filling liquid hydrogen	★★ large initial investment
solid-state hydrogen	solid-state hydrogen storage			
	metal based		in the testing stage, high hydrogen storage density, high security, high development potential	★★★★☆ R&D costs may be high for the moment
	nonmetal based			
	hydrogen hydrate			
raw materials for hydrogen production	ammonia transport		low risk of combustion and explosion, low transportation difficulty, and zero carbon. High cost of production and not convenient for terminal applications	★★★★ large investment in front end and terminal

a The more ★, the lower the cost.

High-pressure hydrogen gas storage and transportation is currently the most widely used method.^16^ Hydrogen is pressurized to a certain pressure by a compressor at ordinary temperature, stored in a gas tank, and then transported to the destination in a sealed container or pipeline for pressure regulation.^17^ At present, the pressures of high-pressure hydrogen gas are usually 15, 35, and 70 MPa. In particular, 15 MPa hydrogen cylinders are already well established, hydrogen refueling stations are currently mainly used at 35 and 70 MPa for storage and transportation, and 70 MPa for hydrogen storage and transportation is a hot spot for research.^18,19^

The density of liquid hydrogen is 845 times higher than the density of hydrogen gas at ordinary temperature and pressure, and the energy density per unit volume is several times higher than that of high-pressure gas hydrogen storage.^20^ Liquefied hydrogen storage is the process of compressing and deep cooling hydrogen to under 21 K to become liquid hydrogen,^21^ which is then stored in a specific adiabatic vacuum vessel. The same volume of hydrogen storage vessel stores a larger amount of liquid hydrogen. Liquid hydrogen storage is a highly desirable form of hydrogen storage if only mass and bulk densities are considered. The principle of liquid hydrogen transportation is similar to that of high-pressure gas hydrogen transportation, with the main difference being the higher insulation requirements for storing and transporting liquid hydrogen.^22,23^ Liquid hydrogen pipeline transportation is currently mainly used in hydrogen production plants or rocket launch sites^24^ using vacuum-jacketed adiabatic hydrogen pipelines for transport. Long-distance liquid hydrogen transportation technology has not yet become practical. Researchers have continuously proposed and optimized zero-boiloff liquid-hydrogen storage based on active refrigeration technology.^25−27^ In LHOC technology, specific organic unsaturated compounds such as olefins, alkynes, and aromatic hydrocarbons are hydrogenated with hydrogen under the action of a catalyst to generate hydrogen bond complexes, thus realizing safe and efficient transportation of hydrogen under normal temperature and pressure.^14,28^ At the transport destination, the complex is dehydrogenated to obtain hydrogen.

**Figure 2:**
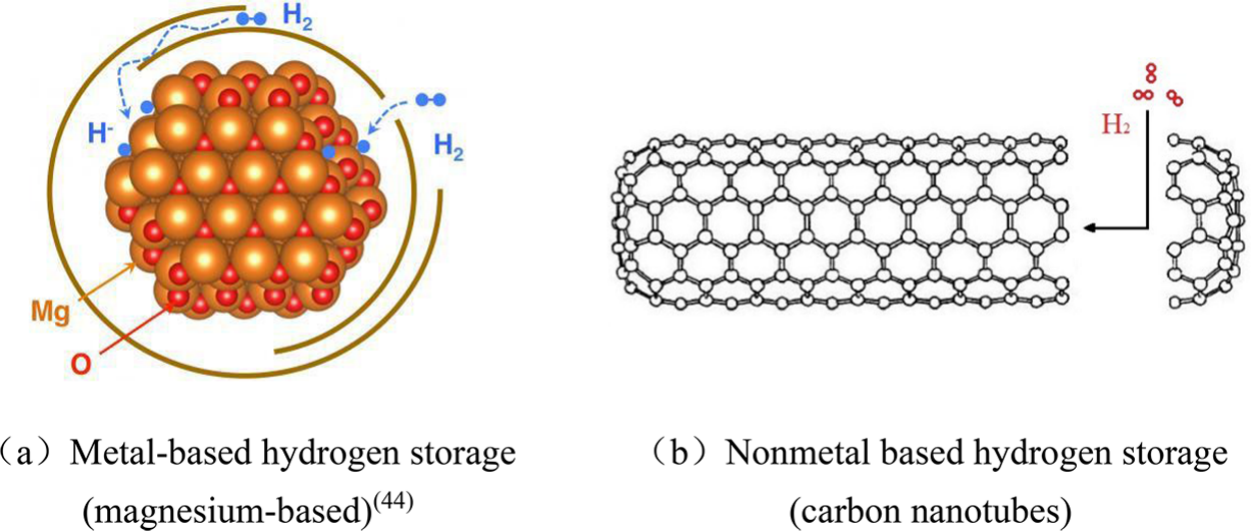
Schematic diagram of solid-state hydrogen storage.

Solid-state hydrogen is stored by using the hydrogen-absorbing characteristics of metals or alloys such as the rare-earth series,^29−31^ titanium series,^32,33^ zirconium series,^34−36^ and magnesium series,^37−39^ as well as nonmetals such as activated carbon and carbon nanotubes,^40−42^ to react with hydrogen to produce stable hydrides (Figure 2). After being transported to the destination under normal temperature and pressure, hydrogen is released by heating. The density of solid-state hydrogen storage even exceeds that of liquid hydrogen storage, which is still in the experimental stage. Under certain pressure and temperature conditions, hydrogen reacts with water to form a cage-like crystal compound. Some hydrogen hydrates have good hydrogen storage performance.^43,44^

Finally, regarding the transportation of hydrogen production raw materials, for example, transporting ammonia to the destination for decomposition to produce hydrogen can effectively avoid the risks that may arise from high-pressure hydrogen and liquid hydrogen.^45−47^ At the same time, policy restrictions based on security considerations may also be implemented.^48^ Using ammonia to store hydrogen has a high hydrogen storage density and can easily achieve liquefaction at lower pressures, further improving the efficiency of transportation.

As the sources of hydrogen are not evenly distributed, it is necessary to transport hydrogen to the corresponding market over a long distance. Although there are various ways of storing and transporting hydrogen, high-pressure gas hydrogen pipeline transportation has significant advantages in efficiency and cost for large-scale centralized hydrogen production and long-distance hydrogen transportation. It is the most economical method and is expected to become the optimal transportation mode. In this article, we will focus on the development trend and problems of hydrogen pipeline transportation according to the development of long-distance hydrogen pipelines worldwide, which can provide a reference for subsequent hydrogen energy transportation.

## 3 Hydrogen Pipeline Transportation

### 3.1 High-Pressure Gas Hydrogen Pipeline Transportation

Pipeline transportation of hydrogen started earlier in developed countries. The most typical case of a hydrogen pipeline^50^ is the 208 km long hydrogen pipeline built and put into operation by the Hull Chemical Plant in the Rhine Ruhr industrial area of Germany in 1939, which is in good operation. According to the “White Paper on China’s Hydrogen Energy and Fuel Cell Industry” published in 2019,^51^ the US already has 2500 km of hydrogen pipelines, Europe has 1598 km of hydrogen pipelines, China’s hydrogen pipelines are still in their infancy, and it is expected that China will build more than 3000 km of hydrogen pipelines by 2030. The relatively short history of the construction of pure or high hydrogen-containing pipelines in China and the relatively large number of medium and low hydrogen-containing pipelines used to transport city water gas provide practical guidance for the subsequent construction of hydrogen pipelines on a large scale. Table 2 is an overview of some long-distance hydrogen pipelines in China.

**Table 2:** Overview of Long-Distance Hydrogen Pipelines in China

project name	length (km)	year of production	pipeline capacity	usage
Baling–Changling hydrogen pipeline	42	2014	50000 tons/year	send raw gas containing 85.84% volume of hydrogen after methanation from Baling Petrochemical’s coal gas plant to Baling Petrochemical’s Yunxi area and Changling Refinery for purification to 99.5% volume of hydrogen
Jinling–Yanzi hydrogen pipeline	17	2007	40000 tons/year	deliver Jinling Petrochemical coal to hydrogen and PSA purified hydrogen to Yangzi Petrochemical for secondary processing
Jiyuan–Luoyang hydrogen pipeline	25	2019	100400 tons/year	deliver Jinling Petrochemical coal to hydrogen and PSA purified hydrogen to Yangzi Petrochemical for secondary processing
Wuhai–Yinchuan coal gas long-distance pipeline	216.4	2012	1.61 billion m^3^/year	coke oven gas from Wuhai Qianli Mountain Industrial Park and Xilai Feng Industrial Park and the hydrogen mixture from the park’s LNG plant and clean oil plant are transported to Ningxia Petrochemical Company
Yima–Zhengzhou coal gas long-distance pipeline	194	2001		deliver high-quality clean coal gas from Yima Mine Pit mouth Gasification Plant to Zhengzhou, Luoyang, Yima and other urban transmission and distribution networks

At present, in terms of engineering applications, basic research and case studies on hydrogen infrastructure transformation and system optimization have received widespread attention, with related technical research mainly focusing on pipeline transmission processes, pipe evaluation, and safe operation assurance.

### 3.2 Hydrogen-Mixed Natural Gas Pipeline Transportation

**Figure 3:**
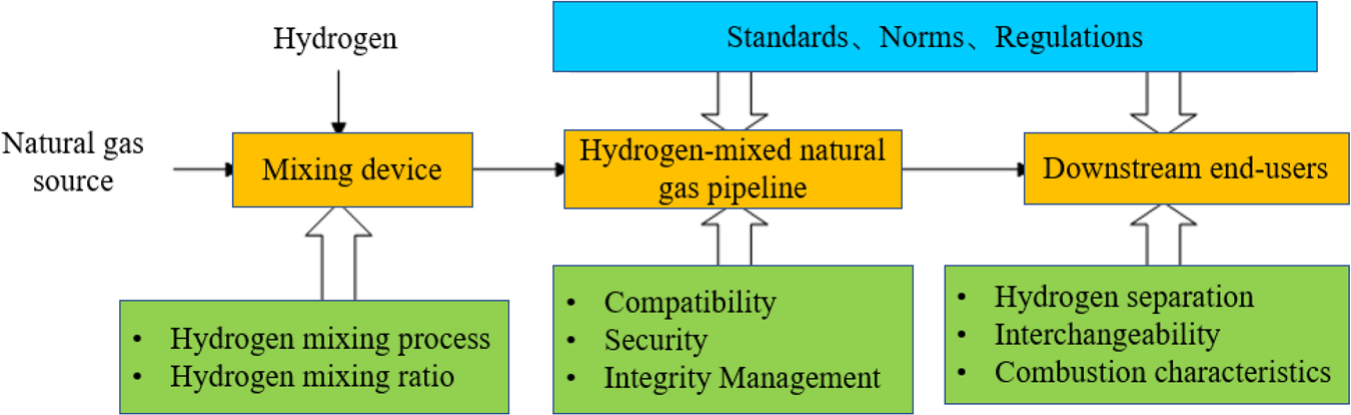
Hydrogen compressed natural gas process diagram.

As a clean fossil energy, natural gas is currently developing rapidly in the world, and there are a large number of natural gas pipelines built and planned in the world. Compared with natural gas pipeline systems, the construction cost of hydrogen transmission pipelines is more than 10% higher.^52^ In recent years, mixing hydrogen into natural gas pipelines for transportation has become a research hotspot of international scholars.^53^ Hydrogen compressed natural gas (HCNG) is also known as Hythan (hydrogen–methane mixture). Through a discussion of the global research status, typical project cases, and problems and advantages of hydrogen blending in natural gas, the study believes that although there are some problems in hydrogen blending in natural gas pipeline networks, it is still the best way to expand the use of hydrogen energy and efficiently transport hydrogen at this stage. The hydrogen compressed natural gas process is shown in Figure 3.

#### 3.2.1 Effect of Hydrogen Mixing Ratio

The existing natural gas transmission and distribution network mainly includes gas storage facilities, long-distance trunk lines, compressor stations, pressure-reducing stations, urban distribution pipelines, and customer terminals. Meanwhile, natural gas spherical tanks, high-pressure bundle storage, end of long-distance pipeline storage, and urban high-pressure pipeline storage also need to be considered,^54^ as these short-term storage methods can effectively solve the problem of urban gas peaking. The impact of injecting hydrogen into a natural gas pipeline covers an extremely wide range of factors. The determination of the hydrogen mixing ratio is a systematic challenge for a variety of factors, such as natural gas composition, pipeline equipment material, hydraulic conditions, facility operating life, environmental impact conditions and differences in customer terminal equipment.^55^ This is still in the research stage, and no uniform standard has been developed. The NATURALHY project study of the European Union believes that safety will not be significantly affected under a hydrogen mixing ratio of 20%, and even a hydrogen mixing ratio of up to 50% is feasible, but it must be evaluated according to the specific situation.^56^ The Sustainable Ameland project in The Netherlands carried out research on the hydrogen mixing ratio for pipeline and household performance, with an average annual hydrogen mixing ratio of 12% in 2010. The GRHYD project funded by the French Environment and Energy Control Agency provides users with hydrogen-mixed natural gas with a mixing ratio of up to 20%, proving the process and economic feasibility.^57,58^ The HeDeploy project in the UK injected hydrogen into the natural gas network at a rate of 20% by volume, demonstrating that the performance and safety of the customer terminal is largely unaffected without changes to the network equipment.^59,60^ In addition, the United States, Germany, Italy, and other countries have also carried out research on the performance and safety impact of natural gas pipeline networks and customer terminals with hydrogen mixing ratios below 20%.^61^ In 2019, the UK’s H21 project opened a 100% hydrogen test facility in Derbyshire with the aim of converting the UK gas network to 100% hydrogen transmission. China’s research on hydrogen mixing of natural gas started late. To promote the process of hydrogen mixing of natural gas, it is necessary to discuss the influence of the hydrogen mixing ratio on different natural gas pipelines and terminal equipment and formulate relevant specifications.

##### 3.2.1.1 Impact on Storage and Transport Facilities

In hydrogen pipelines and steel storage containers, hydrogen molecules tend to react with the metal and cause the pipeline or storage container to fail. A team has carried out research into the compatibility of natural gas pipeline steel with high-pressure hydrogen environments. For X80 pipeline steel material, it was deduced from microscopic observations of tensile results at different pressures and strain rates that the existence of diffused hydrogen near the surface of the steel is the main cause of hydrogen embrittlement. Some scholars^62^ have studied different grades of steel by the electrochemical hydrogen charging method. The research shows that when the hydrogen charging current density reaches a certain limit, the greater the strength of the material, the greater the sensitivity of hydrogen embrittlement. Meng et al.^63,64^ studied the mechanical properties of domestic X70 and X80 pipeline steels mixed with hydrogen in natural gas and found that the yield strength and tensile strength of the two pipeline steels were basically unchanged, but the notch tensile strength was reduced, and the fatigue crack growth rate of the materials was significantly increased. In addition, the effect of natural gas–hydrogen mixtures on steel with hydrogen volume fractions ranging from 0% to 50% at a pressure of 12 MPa was carried out on X80 pipeline steel. With increasing hydrogen content, the plasticity and fracture toughness of the material were significantly reduced, and the fatigue crack growth rate was significantly accelerated. The study of the NATURALHY natural gas hydrogen mixing project in the EU found that the safety and stability of pipeline steels is not significantly affected at 20% hydrogen mixing ratios, and even at 50% hydrogen mixing ratios, no serious hydrogen damage is caused.^56^ Most of the above scholars and teams believe that steel pipes for hydrogen pipelines are preferred to low steel grade steel pipes. Kane et al.^65^ studied the feasibility of transporting hydrogen in polymer pipelines. Hydrogen has little impact on polymer materials such as polyethylene (PE) or polyvinyl chloride (PVC) and has strong compatibility. However, considering the aging of polymer pipelines in the soil and atmosphere and the permeability of hydrogen in polymer pipelines being higher than that of metal pipelines, specific research is still needed in practical applications.

The calorific value of natural gas is approximately 3 times that of hydrogen.^66^ To meet the same energy demand, Huang et al.^67^ believed that increasing the transmission pressure can meet the requirements for gas transmission power. Wang et al.^68^ used HYSYS software to build a model. When the hydrogen ratio is 30% at 15 °C, the pipeline outlet pressure increases by 9.1%, the gas transmission volume increases by 14.8%, and the gas transmission power decreases by 9.2%. The pipeline operating pressure can be increased appropriately to meet the gas transmission power. Wu et al.^69^ conducted a steady-state simulation of hydrogen mixing through hydraulic simulation software, and the analysis showed that the mixing of hydrogen in a natural gas pipeline has little impact on the hydraulic conditions of the pipeline network. The compressor is the main piece of equipment providing pressure for the natural gas pipeline. Wang et al.^68^ found that when the compressor’s fixed speed remains unchanged, compared with the pure natural gas working condition, when the hydrogen mixing ratio is 30%, the compression ratio decreases by 20%, and the shaft power decreases by 36%. For this problem, Haeseldonckx et al.^66^ believed that the transmission pressure can be increased by properly increasing the compressor speed, but most natural gas compressors currently in service do not consider the impact on the blade material under various conditions of hydrogen compression.

In general, there is still a lack of research on the quantitative impact results, damage prediction, and preventive measures of pipeline hydrogen damage to clarify the principle of various mechanisms of hydrogen damage, the synergy between different mechanisms, and the synergistic effects of hydrogen and sulfide, carbon monoxide, and carbon dioxide. The hydrogen mixing ratio has little impact on the hydraulic conditions and compressor performance, but there are great differences between different pipelines and equipment, so it is still necessary to carry out targeted evaluation on different pipelines.

##### 3.2.1.2 Impact on Domestic Gas Installations

**Figure 4:**
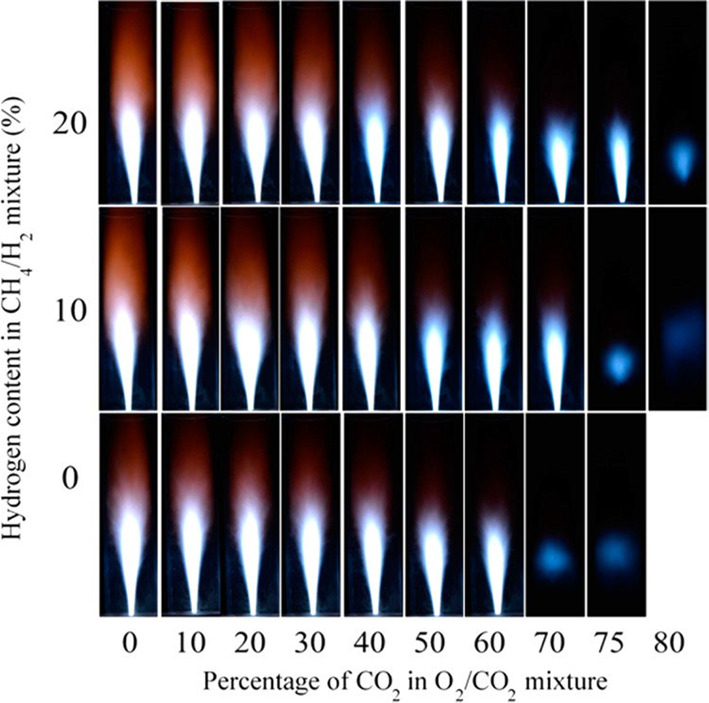
Flame images for different oxidizer (O_2_/CO_2_) mixtures and fuel compositions.^71^

The Wobbe index is obtained by dividing the high calorific value of the gas under the specified reference conditions by the square root of its relative density under the same reference conditions. The higher the Wobbe index of a gas, the greater the calorific value of the amount of gas flowing through a given size hole in a given time. Therefore, it is a measure of gas interchangeability and application applicability. For ordinary rich natural gas burners, the Wobbe index must be between 48 and 58 MJ/Nm^3^. When using lean natural gas, the Wobbe index must be between 41 and 47 MJ/Nm^3^. For lean natural gas burners, up to 98% hydrogen can be added, and for rich natural gas, up to 45% hydrogen can be added.^66^ However, the most important effect of using hydrogen in burners, boilers, or gas engines is the increase in flame speed, which brings the risk of backfire and makes combustion unstable. Schefer^70^ carried out experiments on the combustion of hydrogen–methane mixtures at hydrogen ratios of 0, 0.12, 0.22, and 0.29 and took photographs of the combustion state. As shown in Figure 4, Sanusi et al.^71^ conducted experimental research on the oxygen combustion characteristics of methane and hydrogen-rich methane in a nonmixed swirl stable burner. The experiment showed that with an increase in the hydrogen ratio, the flame length decreased, indicating that the combustion was more rapid. Haeseldonckx et al.^66^ believes that a hydrogen doping ratio below 17% will not cause problems. Ma et al.^72^ analyzed the impact of the hydrogen mixing ratio on the burner thermal load, primary air coefficient, combustion stability, thermal efficiency, and flue gas pollutants. The research showed that with an increase in the hydrogen mixing ratio, the primary air coefficient of the burner increases, the measured heat load decreases, and the content of flue gas pollutants such as CO, NO, and NO_*x*_ decreases. When the hydrogen ratio reaches 20%, the thermal efficiency can be increased by more than 2%. The hydrogen mixing ratio has a great impact on the safety and efficiency of domestic gas, and the quality of domestic gas facilities is different, so much verification is still needed to determine the hydrogen mixing ratio suitable for domestic use. It is noteworthy that a government-provided gas alarm is commonly installed in Chinese apartments, but most of them can only detect methane or carbon monoxide. If hydrogen is added to the city gas, it will diffuse at a higher rate, posing a greater safety threat to these apartments. Moreover, people may not receive a timely warning before an explosion occurs. Some scholars are currently developing new types of sensors, such as MOF-^73^ and polymer-based^74^ hydrogen leak sensors,^75^ to address this issue.

##### 3.2.1.3 Impact on the Gas Turbine and Engine

Industrial gas is the main force of natural gas consumption. In 2019, China’s natural gas industrial gas consumption accounted for 35.0%, second only to 37.2% of urban gas. Gas turbines are the most important industrial gas facilities. In urban gas, in addition to domestic gas, CNG vehicles are the main consumer end. Many scholars have studied the influence of the hydrogen mixing ratio of gas turbines and engines. Schefer et al.^70^ carried out tests on gas turbines with different hydrogen mixing ratios. With an increase in the hydrogen ratio, the lean burn limit is reduced, the CO emission is significantly reduced, the concentration of hydroxyl radicals is significantly increased, and the stability is improved. The addition of hydrogen has little effect on the equilibrium adiabatic flame temperature. At a higher equilibrium adiabatic flame temperature, the flame size and shape of hydrogen-rich fuel are similar to those of a methane flame. In 2016, Mitsubishi successfully tested a large gas turbine for power generation using a 30% hydrogen fuel mixture. Stable combustion can be achieved by using the special combustion chamber newly developed by Mitsubishi Hitachi. Compared with pure natural gas power generation, hybrid combustion achieved a 10% reduction in carbon dioxide. Nagalingam et al.^76^ carried out experimental research on an AVL single-cylinder engine, and the results showed that with an increase in the hydrogen mixing ratio, the maximum power of the engine decreased compared with that when methane was used as fuel, mainly due to the lower volumetric calorific value of hydrogen compared to methane, but the faster combustion rate of hydrogen led to a reduction in the optimum ignition advance angle, which helped to reduce NO_*x*_ emissions. Fulton et al.^77^ conducted a study on the combustion lean limit characteristics of the GM 5.7LV8 engine with different hydrogen mixing ratios. The results show that when the hydrogen mixing ratio is 15% and 30%, it can burn in a lower air–fuel ratio environment while reducing NO_*x*_ emissions. When the hydrogen ratio is 30%, the lean burn limit can be widened without reducing the power and economy. Forest et al.^78^ investigated the effects of hydrogen mixing ratios on engines in depth and showed that the best comprehensive engine performance in terms of power, emissions, and economy can be achieved at 20–30% doping ratios, while high doping ratios may lead to engine bursts, reduced power, and increased fuel costs, and low mixing ratios do not have a significant effect on engine performance.

The above studies have positive implications for the use of gas turbines and engines with hydrogen-mixed natural gas and put forward requirements for the ratio of hydrogen to natural gas. However, most researchers have only conducted short-term studies on the effects of hydrogen mixing ratios on certain models or types of gas turbines or engines. The existing gas turbines and engines on the market are different, and there is still a need for more detailed studies on the effects of hydrogen mixing ratios to determine a more universal ratio. At the same time, it provides guidance for the equipment selection and equipment design and production of the newly built hydrogen-mixed natural gas pipeline network.

#### 3.2.2 Hydrogen Separation and Purification Technologies

Direct use of hydrogen-mixed natural gas as fuel can effectively reduce carbon emissions. However, considering some occasions where high-purity hydrogen is used as fuel, such as hydrogen fuel cells and pure hydrogen fuel vehicles,^79^ it is still necessary to develop a process to efficiently separate hydrogen from hydrogen-mixed natural gas. Currently, the available hydrogen separation methods include pressure swing adsorption, membrane separation, electrochemical hydrogen separation, and deep cooling separation.

##### 3.2.2.1 Pressure Swing Adsorption

**Figure 5:**
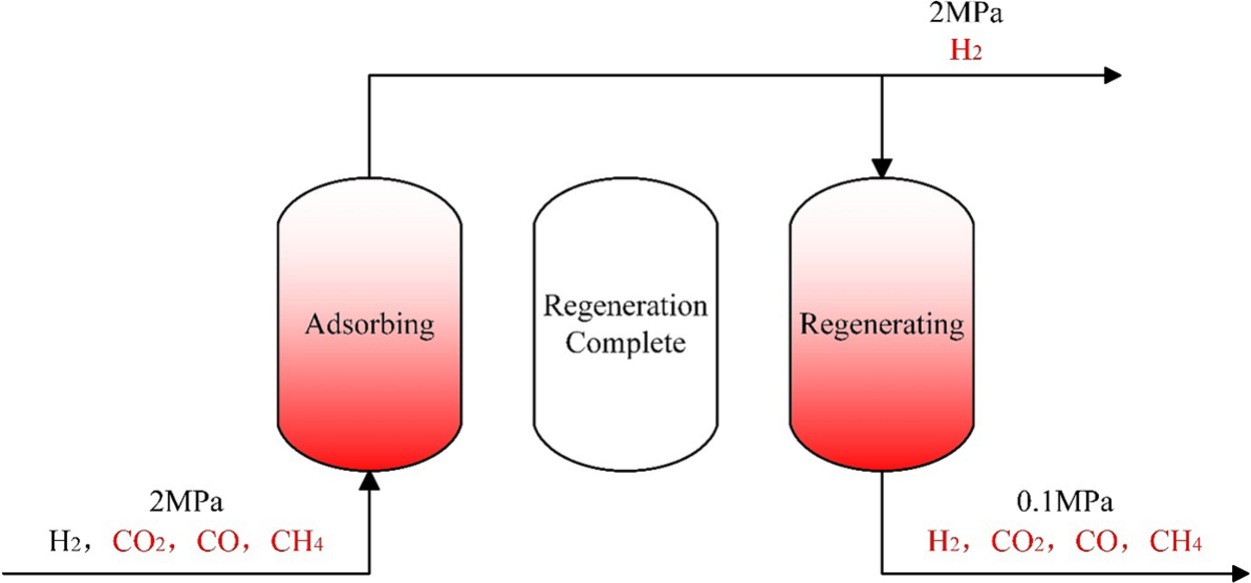
Schematic diagram of the pressure swing adsorption process.

Pressure swing adsorption is a relatively mature technology with a short separation cycle and high separation purity.^80^ The system is usually produced at a scale of 50–200000 N m^3^/h. Pressure swing adsorption operates according to the principle of the adsorption isotherm, and each material has a characteristic correlation between gas surface adsorption and gas partial pressure. With increasing gas pressure, the concentration of adsorbed (fixed) substances on the surface increases. In the adsorption bed, using highly porous adsorption materials, nonhydrogen compounds can be adsorbed under high pressure. Multilayer fillers of different materials are usually used and adjusted to the specific gas composition entering the bed. When reforming gas flows through the packed bed, carbon dioxide, carbon monoxide, methane, and other impurities are adsorbed, while hydrogen flows through the packed bed. When the adsorption bed is saturated, the gas flow will be directed to the newly regenerated bed, and the saturated bed will be regenerated at the same time. In the regeneration stage, the pressure in the container decreases so that the gas adsorbed on the surface returns to the gas phase, and hydrogen can also be used as a purging gas to remove impurities. Pressure swing adsorption can purify hydrogen to 99.97%.^81^ A schematic diagram of the pressure swing adsorption process is shown in Figure 5.

The size of the pressure swing adsorption bed is strongly affected by the concentration of nonhydrogen substances entering. If the impurity concentration is doubled, the pressure swing adsorption bed will need almost twice the saturated part. When lower concentrations of hydrogen are separated, the concentration of nonhydrogen species increases. This means that more gas will be compressed to recover less hydrogen. The hydrogen separation of hydrogen-mixed natural gas can make full use of the pipeline pressure to enter the adsorption bed. However, due to the low hydrogen content, repeated adsorption is required to meet the purity requirements, increasing energy consumption and process complexity,^82^ so further targeted research is needed.

##### 3.2.2.2 Membrane Separation

**Figure 6:**
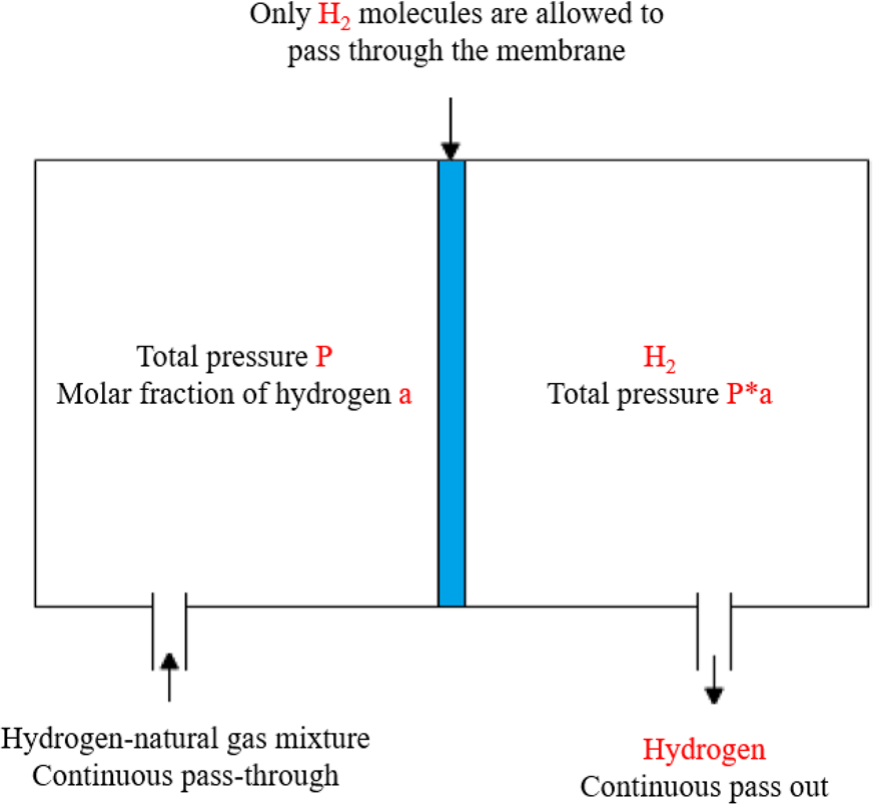
Schematic diagram of hydrogen membrane separation.

This technology is based on the principle of selective permeation, by which the random movement of molecules across the permeable membrane will equilibrate to equal partial pressures on each side of the membrane.^83^ During the separation of the hydrogen membrane, hydrogen is continuously extracted from the pure hydrogen side, making the hydrogen on both sides of the membrane produce a partial pressure difference as the driving force and continue to permeate to the pure hydrogen side.^84^ The process diagram of hydrogen purification by membrane separation is shown in Figure 6.

Membrane separation technology is very effective when the hydrogen concentration is relatively high. Most applications using membrane technology recover a large amount of hydrogen with a purity of 95–99% in industry. The membranes used for hydrogen separation include ceramic membranes, metal membranes, molecular sieve membranes, polymer membranes, etc.^85^ Palladium-based membranes are mostly used for the preparation of high-purity hydrogen, but the cost is high. For hydrogen separation of low-concentration hydrogen-doped natural gas, the large pressure difference between the two sides of the membrane easily causes membrane pressure breakage. To solve this problem, some scholars have proposed a series of supported membranes, such as porous Vycor glass supports,^85^ multihollow ceramic supports,^86^ and multihollow metal supports,^87^ which can withstand large pressure differences.

##### 3.2.2.3 Electrochemical Hydrogen Separation

**Figure 7:**
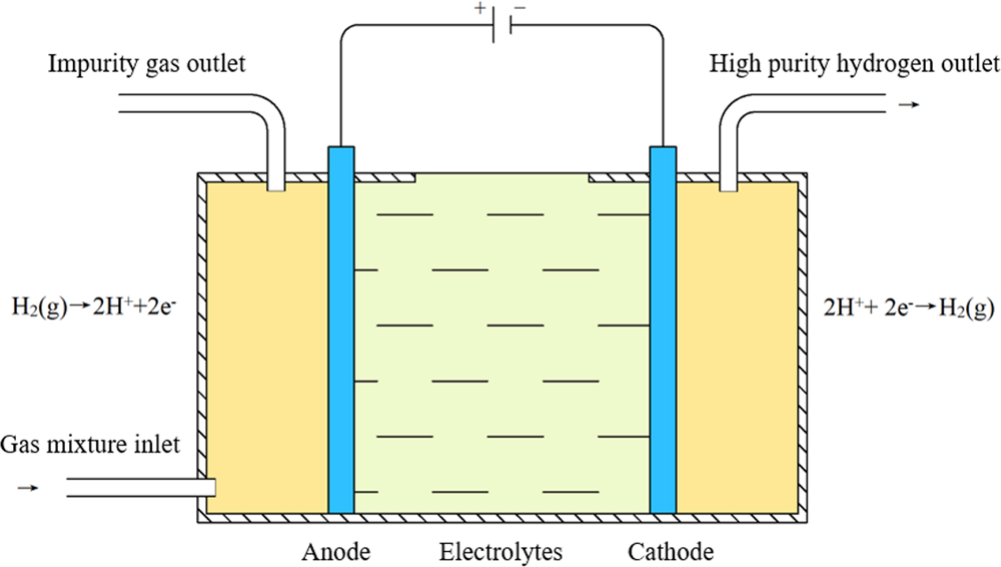
Schematic diagram of electrochemical hydrogen separation.

The working principle is the same as that of the fuel cell system. The fuel cell is used to make the mixed gas pass through one side of the fuel cell and apply current to the cell.^88−90^ The hydrogen atom loses electrons in the anode reaction to form hydrogen ions. The hydrogen ions move to the cathode side under the drive of the electrode to obtain electrons and recombine into hydrogen. A schematic diagram of electrochemical hydrogen separation is shown in Figure 7. The electrochemical hydrogen separation process can effectively separate even when the hydrogen content is very low.

##### 3.2.2.4 Deep Cooling Separation

Deep cooling separation,^91,92^ also known as low-temperature distillation, is a gas liquidation and purification technique invented by Linde in 1902. After the mixed gas is liquefied, the different gases are separated by distillation using the difference in boiling points of the different gases. It is now widely used for the separation of oxygen from air and for the separation of crude oil cracking gas. Under standard conditions, the boiling points of hydrogen and methane are −252.8 and −161.5 °C, respectively. The mixture can be cooled to below −161.5 °C by cooling. At this time, methane is liquefied and separated to obtain high-purity oxygen and liquefied natural gas. However, the liquefaction process consumes a large amount of energy, and its economic feasibility needs to be further verified.

Through the investigation of the above four hydrogen separation processes, it can be found that most of the processes are suitable for separation with high hydrogen content. Since the hydrogen mixing ratio in hydrogen-mixed natural gas is usually lower than 20%, the separation cost of various methods is generally high, and the optimization of separation efficiency for low hydrogen content needs further study.

#### 3.2.3 Current Status of the Hydrogen-Mixed Natural Gas Pipeline

By the end of 2019, according to IEA data, 37 demonstration projects around the world were studying hydrogen mixing in natural gas networks. Table 3 shows typical cases of hydrogen mixing projects in some natural gas distribution networks.

**Table 3:** Typical Project Cases of Hydrogen Mixing in a Natural Gas Distribution Network

date	project name/participants	nation	project introduction
2004–2009	NATURALHY	EU	the impact of natural gas hydrogen mixing on the durability of pipeline networks, the management of the integrity of natural gas pipeline hydrogen mixing, the impact of natural gas hydrogen mixing on safety aspects, the impact of natural gas hydrogen mixing on consumers and end-use equipment, the separation of hydrogen from hydrogen/natural gas mixtures, and its impact on the quality of the remaining gas were studied
2008–2011	Sustainable Ameland	The Netherlands	first long-term test of the performance of hydrogen-mixed natural gas for domestic use, comparison of hydrogen-rich natural gas pipelines and pure natural gas pipelines under the same conditions in the tourist town of Ameland Nes to study the effect of hydrogen-rich natural gas on the pipelines
2013	Falkenhagen	Germany	a demonstration plant with 2 MW of power-to-hydrogen capacity was fully commissioned in 2013, and the hydrogen produced is fed directly into a natural gas pipeline
2014–2018	GRHYD	France	it was completed in the suburb of Dunkirk, and hydrogen was injected into the natural gas network to provide gas fuel consisting of hydrogen and natural gas to 100 residential buildings; the goal is to judge the technical, economic, environmental, and social relevance of technology.
2017–2023	HyDeploy	UK	HyDeploy is the first demonstration project in the UK to inject hydrogen into the gas network, the overall goal is to provide a safety case for hydrogen–natural gas mixing and promote the removal of regulatory barriers required to start the hydrogen mixing market
2017	Gazprom	Russia	study how to mix hydrogen in the existing natural gas pipeline network to deliver the mixture containing up to 20% hydrogen to the European continent.
2019	H21	UK	aims to support the conversion of UK natural gas network to carry 100% hydrogen
2019	Chaoyang renewable energy hydrogen mixing demonstration project	China	fill in the gaps in China’s natural gas pipeline hydrogen mixing specifications and standards and verify the key technologies of the hydrogen “production, storage and transportation, blending and comprehensive utilization” industrial chain
2020	research, development ,and application demonstration of key technologies for hydrogen mixing in natural gas	China	a hydrogen production plant in Zhangjiakou is expected to produce approximately 1,000 tons of hydrogen per year, after purification, it will be transported outward in three directions, one of which will be mixed with the Zhangjiakou municipal gas network and used for domestic cookers and HCNG vehicles

In summary, the study of the natural gas pipeline network hydrogen mixing project includes the impact of the gas mixing ratio on pipelines, network equipment, materials and terminal equipment, the integrity management of gas pipeline hydrogen mixing transport, the impact of gas pipeline hydrogen mixing transport on safety aspects, and the separation of hydrogen from the hydrogen gas mixture and its impact on the quality of the remaining gas. In recent years, much research has been conducted on the pipeline transmission conditions and gas performance of hydrogen-mixed natural gas pipelines. However, most of the research results are not universal, and the conclusions are inconsistent. A large amount of research work is still needed before the large-scale commercial application of hydrogen-mixed natural gas.

## 4 Conclusion

The international hydrogen energy industry is gaining momentum. In general, the key technologies of pure hydrogen and hydrogen-mixed natural gas pipeline transportation are relatively mature and have reached the conditions and level of industrial application. Based on the research and analysis in this paper, the following conclusions and suggestions are proposed. The following conclusions and suggestions are proposed.

(1) There are many existing hydrogen storage and transportation methods, but for large-scale hydrogen transportation, pipeline transportation has significant advantages in terms of efficiency and cost. The construction cost of a pure hydrogen pipeline is high, and hydrogen-mixed natural gas pipeline transportation becomes the best solution for hydrogen pipeline transportation at this stage. The impact of hydrogen on natural gas pipeline materials, transmission facilities, and customer terminals is extremely broad, so interchangeability and compatibility demonstration and hydrogen mixing test run evaluation should be carried out to determine the appropriate hydrogen mixing ratio before realizing natural gas mixing and transmission.
(2) Most hydrogen separation processes are suitable for separation at high hydrogen contents. Since the hydrogen mixing ratio in hydrogen-mixed natural gas is usually lower than 20%, the separation cost of various methods is generally high, and the efficiency optimization of the separation for low hydrogen content needs further study.
(3) To further explore hydrogen energy applications, it is necessary to develop more efficient, low-cost, and low-energy storage materials. Metal hydrides, carbon-based materials, and hydrogen hydrates are safe and efficient ways to store hydrogen, but the hydrogen storage materials that can be industrially produced and applied are not yet mature.
